# Essentialist beliefs about accented speakers moderate the effect of processing fluency on employability ratings

**DOI:** 10.3389/fpsyg.2026.1834790

**Published:** 2026-05-19

**Authors:** Cesar Teló, Mary Grantham O’Brien, Pavel Trofimovich

**Affiliations:** 1 Department of Education, Concordia University, Montreal, QC, Canada; 2 Department of Linguistics, Simon Fraser University, Burnaby, BC, Canada; 3 School of Languages, Linguistics, Literatures and Cultures, University of Calgary, Calgary, AB, Canada

**Keywords:** accent beliefs, accent bias, comprehensibility, employability, essentialist beliefs, gay speech, lay theories, processing fluency

## Abstract

Accented second language (L2) speakers are frequently evaluated less favorably than first language (L1) speakers in employment contexts. Processing fluency (the subjective ease of understanding a speaker) has been identified as an experiential variable that helps explain why L2 speakers tend to receive lower workplace-relevant evaluations. However, less is understood about how processing fluency affects downstream judgments like speaker employability or competence. Drawing on the concept of lay theories, we examined whether listeners’ essentialist beliefs about accented and gay-sounding speakers moderated the relationship between processing fluency and employability judgments. We present a *post hoc* analysis of data from 192 listeners who rated the employability of gay- and straight-sounding L1 and L2 speakers of English. Listeners who more strongly endorsed beliefs that accents are diagnostic of speaker characteristics and amenable to speaker control showed a stronger association between processing fluency and employability ratings. The effect of accent beliefs was most pronounced when the speaker was difficult for listeners to understand, in which case stronger essentialist beliefs were associated with particularly low employability ratings. Essentialist beliefs about gay-sounding men did not moderate the fluency–employability relationship, consistent with the absence of reliable differences in listeners’ processing fluency with gay- and straight-sounding speakers in this sample. We provide exploratory evidence that lay theories of what accents reveal about speakers help explain when and for whom processing fluency might affect employment-relevant judgments.

## 1 Introduction

Unfair treatment based on how a person sounds is pervasive, affecting speakers of stigmatized varieties such as second language (L2) and gay-sounding speakers who are frequently downgraded in evaluations compared to normative-sounding speakers (Fasoli et al., 2017; Spence et al., 2024). For example, gay and gay-sounding men are often perceived as warm and socially skilled (Fasoli and Maass, 2020; Hajek and Giles, 2005), yet also as less competent and moral than straight-sounding men (Fasoli et al., 2017), and are downgraded on leadership- and management-relevant traits (Liberman and Golom, 2015; Taylor and Raadt, 2021). Accented L2 speakers similarly elicit biased workplace evaluations (Roberts, 2021), where they are often judged as less competent or intelligent than first language (L1) speakers (Hosoda et al., 2007; Roessel et al., 2018, 2019; Russo et al., 2017).

Listener biases generally rely on socially learned associations between vocal cues and trait inferences that link certain ways of speaking with warmth, competence, or authority (Kinzler, 2021). Primary origins of bias are therefore in social categorization and stereotyping, where listeners use a speaker’s vocal cues to infer their group membership and then extend stereotypical beliefs about this group to the speaker, with negative consequences for speaker evaluations (Fiske and Neuberg, 1990; Kunda and Thagard, 1996; Ryan, 1983). Another pathway to bias is experiential (Alter and Oppenheimer, 2009), whereby listeners who experience subjective processing difficulty (disfluency) with a speaker’s utterance tend to report negativity (e.g., frustration) and consequently downgrade the speaker in their evaluations (Dragojevic and Giles, 2016; Dragojevic et al., 2017). Current frameworks of accent bias thus emphasize parallel, additive contributions of stereotype content and processing fluency to language attitudes (Dragojevic and Dayton, 2025; Dragojevic et al., 2025).

In a recent article (Teló et al., 2026), we similarly found separate stereotype-driven and processing-fluency contributions to listener judgments of employability for gay- and straight-sounding L1 and L2 speakers evaluated through audio-recorded responses to an interview question. Besides employability, listeners judged the speakers’ sexual orientation and assessed their processing fluency (i.e., comprehensibility defined as ease of understanding). Straight-sounding L1 speakers received the highest employability ratings, followed by straight-sounding L2 speakers and gay-sounding L1 speakers, with gay-sounding L2 speakers ranked lowest. Importantly, processing fluency mediated the relationship between a speaker’s language status (but not sexual orientation) and employability, where L2 speakers were rated less employable in part because they were more difficult to understand, with processing fluency explaining approximately 50% of the variance in employability.

Less clear, however, are the mechanisms linking listeners’ processing fluency to their judgments about speakers. One way in which processing fluency can become relevant to listeners is top-down, as listeners interpret their subjective experience with a speaker’s utterance through their beliefs, assumptions, or lay (naïve) theories about their own or the speaker’s intentions and skills (Dragojevic and Giles, 2016; Winkielman et al., 2025). For example, Dragojevic and Gasiorek (2023) demonstrated that listeners who received positive feedback about their test performance featuring a strongly accented L2 speaker reported greater processing fluency and more favorable evaluations of the speaker. Similarly, Montgomery and Acheme (2022) reported an association between listeners’ processing fluency and the intentions they attributed to the speaker (e.g., being more or less willing to speak effectively and clearly), which in turn predicted listeners’ judgments of speaker competence and friendliness. Thus, listeners’ conceptually driven expectations about their own performance or their top-down interpretations of speaker intentions may explain how listeners’ processing fluency is related to their social judgments about speakers.

Another example of listeners’ top-down, interpretive assumptions about the speaker, with potential consequences for how they interpret their experience of processing fluency, includes essentialist beliefs. Essentialism refers to the idea that socially constructed groups possess natural, deep, and stable traits, and that members of these groups share an underlying “essence” that explains their qualities and behaviors (Haslam et al., 2002). For example, nationality and sexual orientation are often treated as biologically based or fixed early in life (Bastian and Haslam, 2008; Haslam and Levy, 2006), and immigrants and gay men are viewed as sharing core similarities with other members of their respective groups, forming bounded categories (Lytle et al., 2017; Zagrean et al., 2024). Such essentialist beliefs can also be used to rationalize a person’s membership in a social group, such as why some men are gay, and explain traits associated with a group, such as why immigrants are perceived as threatening (Haslam and Levy, 2006; Peherson et al., 2009).

As a cue to social categories, a speaker’s accent can be the focus of essentialist beliefs. Stigmatized accents are often believed to reflect a speaker’s inherent and stable characteristics, such as being non-white or from someplace else, and to provide diagnostic information about the speaker, such as having competence and goodwill (Dragojevic et al., 2016; Subtirelu, 2015; Shuck, 2006). According to Hansen (2020), essentialist beliefs about accents can be captured through the perceived dimensions of diagnosticity (the extent to which accents reflect broader speaker traits) and controllability (the extent to which accents are difficult to change, including through effort), which in turn inform listener evaluations of accented speakers. For instance, if listeners believe that accented speakers can eliminate their accents through effort or choice, listeners tend to ascribe harsher judgments to accented speakers (Hansen, 2020). Similar processes may apply to gay-sounding speakers, because vocal cues associated with sexual orientation can be construed as revealing underlying differences between gay and straight people (Gaudio, 1994; Vasilovsky, 2018). Fasoli et al. (2021) documented essentialist beliefs about gay-sounding speakers along the perceived dimensions of discreteness (the extent to which gay and straight people have categorically different voices), immutability (the extent to which differences between gay- and straight-sounding people are deep-rooted and fixed), and controllability (the extent to which speakers choose to conceal or emphasize their sexual orientation through pronunciation). As with accent beliefs, if listeners believe that gay-sounding speakers can manipulate how gay they sound, listeners tend to provide less favorable judgments to those speakers (e.g., Fasoli et al., 2021; Kiebel et al., 2020).

## 2 The current study

If essentialist beliefs represent another source of top-down, conceptually driven assumptions that listeners use to interpret their experience of processing fluency (Schwarz et al., 2021; Winkielman et al., 2025), then these beliefs might moderate the relationship between listeners’ processing fluency and their evaluations of speakers (Dragojevic and Giles, 2016; Montgomery and Acheme, 2022). To explore this possibility, we conducted a *post hoc* analysis of our previous dataset (Teló et al., 2026), examining whether listeners’ essentialist beliefs about L2 speakers and gay men (collected through questionnaires after the rating) moderate the extent to which listeners’ processing fluency predicted their judgments of speaker employability. Our exploratory question was: Do listeners’ essentialist beliefs moderate the relationship between listeners’ processing fluency and their judgments of speaker employability?

Because essentialist beliefs about accented L2 speakers reflect assumptions that accent-related characteristics are stable and informative of a speaker’s underlying qualities (Hansen, 2020), listeners who more strongly endorse such beliefs may be more likely to interpret processing disfluency (i.e., moment-to-moment experience of difficulty in understanding) as reflecting enduring and potentially negative or non-conforming characteristics of the speaker. Therefore, we expected that the relationship between processing fluency and employability would be stronger among listeners who more strongly endorsed essentialist beliefs about L2 speakers (Montgomery and Acheme, 2022). Following similar reasoning, at least in principle, essentialist beliefs about gay-sounding men could also interact with listeners’ experience (Unkelbach and Greifeneder, 2013; Winkielman et al., 2025), assuming that the gay-signaling voice cues that activate gender or sexuality stereotypes for the listener might also disrupt processing fluency (Mack and Munson, 2012; Sulpizio et al., 2025; Sumner et al., 2014). However, because processing fluency did not mediate the relationship between a speaker’s sexual orientation and employability in our dataset (Teló et al., 2026), we did not anticipate the relationship between processing fluency and employability to be differentiated through listeners’ essentialist beliefs about gay-sounding men.

## 3 Methods

### 3.1 Listeners

We recruited 192 listeners (*M*_age_ = 34, *SD* = 12, range = 18–71), all residents of Calgary (Canada), using census data (Statistics Canada, 2023) to approximate the proportion of Calgarians born in Canada (64% in the sample; 65% in Calgary) and outside Canada (36% in the sample; 33% in Calgary). The sample, which was otherwise self-selected, included 62% self-identified women, 35% men, and 3% non-binary people. In terms of sexual orientation, 70% were heterosexual, 27% identified as asexual, bisexual, gay, lesbian, or queer, and 3% chose not to disclose. As a group, 37% hailed from Calgary, 27% from another Canadian city, and 36% from outside Canada. Listeners reported varying levels of multilingualism (57% spoke one language, 31% two, 9% three, and 3% four) and identified English as the most common L1 (69%), followed by English plus another language (4%), Spanish (4%), and 22 other languages (23%). For additional listener background details, including their racial prejudice and homonegativity, (see Appendix A in Supplementary material).

### 3.2 Recordings and procedures

The stimuli consisted of audio recordings (*M* = 41 seconds) of job candidates responding to the question: “What makes you a good candidate for this job?” Four scripted scenarios were used, each for a different occupation varying in communication demands (high vs. low) and occupational stereotypicality (gay-typed vs. straight-typed). The four occupations were bus driver (low communication, straight-typed), school principal (high communication, straight-typed), fashion designer (low communication, gay-typed), and flight attendant (high communication, gay-typed). Norming studies confirmed that the occupations differed as intended in these dimensions while being similar in perceived response quality. The scenarios were comparable in length (approximately 120 words) and content structure. The recordings were produced by eight men: two straight- and two gay-sounding L1 English speakers, and two straight- and two gay-sounding L1 Spanish speakers who spoke L2 English. The speakers recorded 32 files (4 scenarios × 8 speakers), organized in 16 balanced experimental lists, each containing four target recordings for a within-participant, sparse rating design, with 8–16 unique listeners per list.

Listeners completed the study online through Qualtrics, with one practice trial followed by four experimental trials presented in random order. On each trial, listeners heard a recording of a job candidate twice. After the first playback, they rated the speaker’s accentedness (the extent to which the speaker sounded non-native; 1 = *heavily accented*, 100 = *not accented at all*) and comprehensibility as a measure of listeners’ processing fluency (the degree of difficulty in understanding the speaker; 1 = *very hard to understand*, 100 = *very easy to understand*). After a second playback, they rated the speaker’s employability (likelihood of hiring the candidate; 1 = *not at all*, 100 = *very much*). Additional scales targeted perceived sexual orientation, job prestige, and personality traits. Further information about stimulus development, validation, and participant recruitment are available in Teló et al. (2026) and Supplementary material.

### 3.3 Essentialist beliefs

After rating, listeners completed two 6-item scales assessing essentialist beliefs about accented speakers and gay-sounding men (see Appendix B in Supplementary material). For accent, adapted from Hansen (2020), the items targeted beliefs about accent diagnosticity (whether a speaker’s accent is diagnostic of other speaker traits) and controllability (whether a speaker’s accent is stable or otherwise malleable). For gay-sounding voice, adapted from Fasoli et al. (2021), the items targeted voice-based beliefs about discreteness (whether gay and straight speakers sound categorically different), immutability (whether pronunciation differences between gay and straight men are deep-rooted and fixed), and controllability (whether speakers choose to conceal or emphasize their sexual orientation through pronunciation). All items were rated on a 7-point scale (1 = *strongly disagree*, 7 = *strongly agree*) and showed acceptable consistency across the items targeting accented speakers (Cronbach’s α = 0.77) and gay-sounding men (α = 0.69), so a composite score was calculated per listener by averaging all relevant scale items. Higher accent essentialism scores indicated stronger beliefs that a person’s accent (type and strength) is an important, informative trait and that accented speakers can change or eliminate their accents. Higher gay-sounding essentialism scores indicated stronger beliefs that sounding gay is determined early in life, can be easily detected, and that gay-sounding men choose to sound stereotypically gay.

### 3.4 Data analysis

Analytically, we followed our previous report testing processing fluency as a mediator of the relationship between speakers’ social membership (L1 vs. L2, gay vs. straight) and their employability (Teló et al., 2026). Here, we examined a boundary condition of this relationship, namely, whether essentialist beliefs moderate the extent to which processing fluency predicts employability. We used linear mixed-effects models (Gaussian distribution) to account for the nested data structure, with random intercepts for listeners and speakers. For stronger analyses, we combined the data across job types, which differed in communication demands and occupational stereotypicality. For the final model, visual inspection of the residuals indicated good overall agreement with the expected distribution, with small tail departures confirmed through a Kolmogorov-Smirnov goodness-of-fit test (*p* = 0.001), suggestive of mild heteroskedasticity. Dispersion was not considered problematic (*p* = 0.896). Thirteen outliers were observed but not removed from the dataset because this number fell within the expected outlier frequency (*p* = 0.080). Given the outcome variable (employability assessed through a 100-point scale), we treated these diagnostics as indicating acceptable model specification.

## 4 Results

**FIGURE 1:**
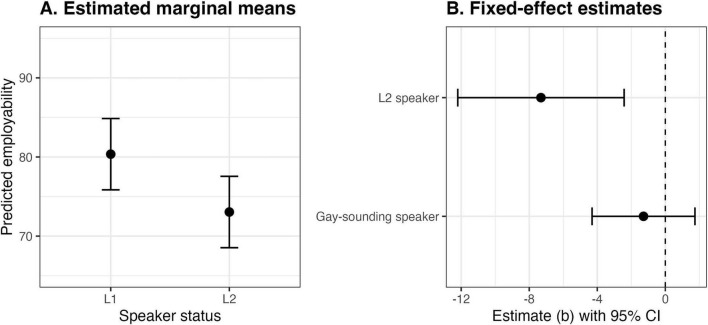
Estimated marginal means and 95% CIs for speaker language status **(A)** and fixed-effect estimates and 95% CIs **(B)**, with reference categories omitted for clarity.

**FIGURE 2:**
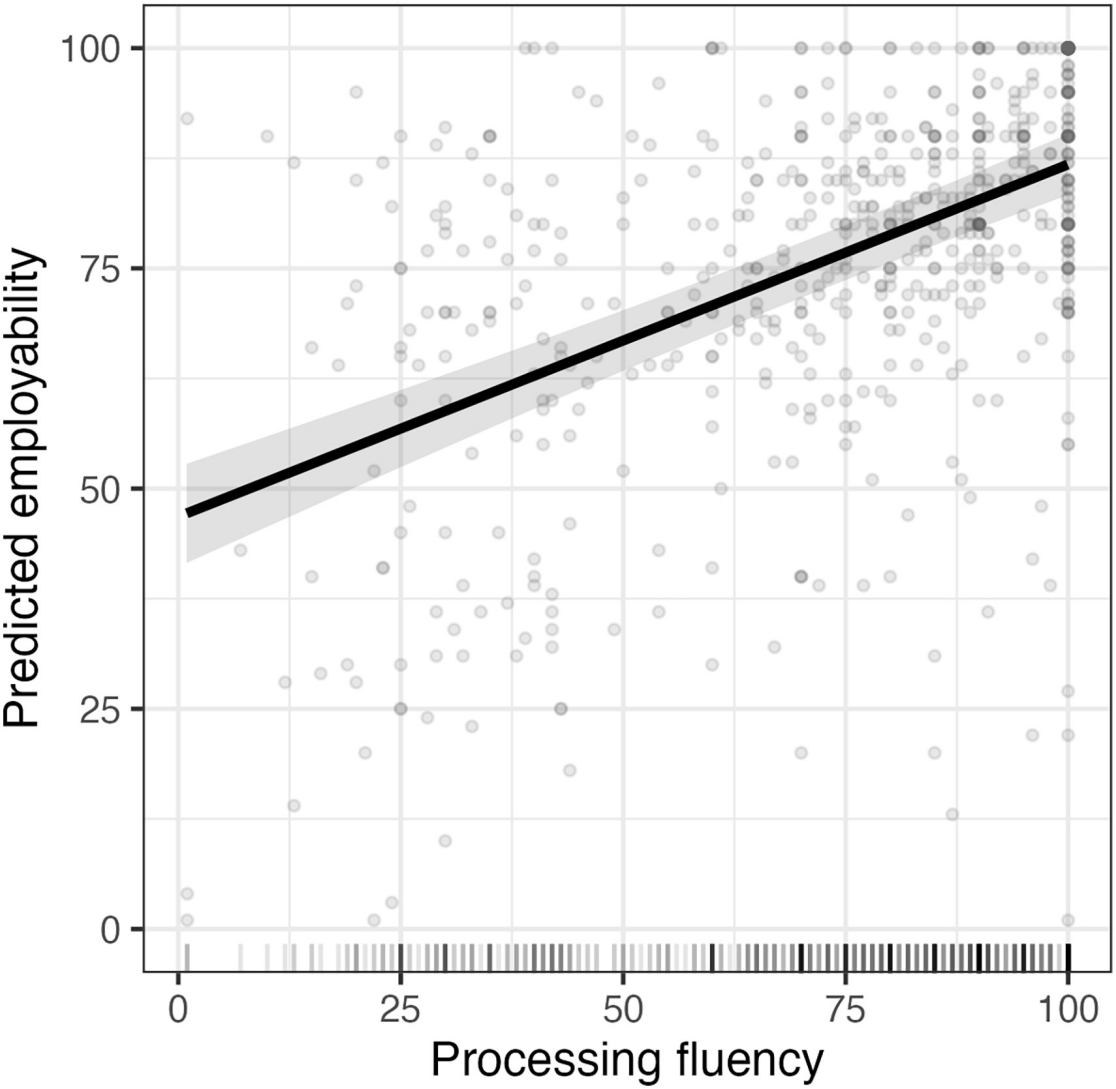
Predicted employability (and 95% CIs) as a function of processing fluency.

We first fit a model with fixed effects for speaker language status and sexual orientation (both binary). This model, illustrated in Figure 1, explained 3% of variance in employability (*R*^2^_conditional_ = 0.43), with L2 speakers rated as less employable than L1 speakers, *b* = –7.31, *SE* = 2.49, *t* = –2.93, *p* = 0.028. Updating this model with listener ratings of accentedness (Δ*R*^2^_marginal_ = 0.03) and processing fluency (Δ*R*^2^_marginal_ = 0.17) and resulted in 23% of the variance explained (*R*^2^_conditional_ = 0.52). As a group, listeners rated the L1 speakers (*M* = 77.44, *SD* = 27.01) as less accented than the L2 speakers (*M* = 36.30, *SD* = 24.42), *t*(6) = 13.34, *p* < 0.001. Listeners also demonstrated greater processing fluency with the L1 speakers (*M* = 85.08, *SD* = 19.54) than the L2 speakers (*M* = 63.37, *SD* = 25.28), *t*(5.60) = 7.19, *p* < 0.001, with accentedness and processing fluency correlated at ρ = 0.63, consistent with a strong association (Plonsky and Oswald, 2014). Greater processing fluency (perceiving a speaker as easier to understand) was associated with higher employability, *b* = 0.40, *SE* = 0.03, *t* = 12.02, *p* < 0.001, as shown in Figure 2, but accentedness did not reliably predict employability, *b* = –0.02, *SE* = 0.03, *t* = –0.76, *p* = 0.446.

We then modeled listeners’ essentialist beliefs. Listeners exhibited moderate levels of essentialist beliefs about accented speakers, at a mean of 3.65 (*SD* = 1.23, range = 1.00–6.83), and about gay-sounding men, at a mean of 3.75 (*SD* = 1.09, range = 1.00–7.00), both on a 7-point scale. The two beliefs scores correlated at *r* = 0.55, 95% CI [0.50, 0.61]. Including listeners’ essentialist beliefs as main effects improved the baseline model’s fit, χ^2^(2) = 8.59, *p* = 0.014, and explained an additional 2% of employability ratings. Addressing our main question, two-way interaction terms between listeners’ processing fluency and essentialist beliefs similarly improved model fit relative to the main-effects model, χ^2^(2) = 11.18, *p* = 0.004, and explained an additional 1% of employability ratings, for a final model explaining 26% of the variance (*R*^2^_conditional_ = 0.54; 620 valid datapoints). We also examined whether the moderating role of essentialist beliefs depended on speakers’ status (L1 vs. L2, gay vs. straight). Higher-order interactions were tested, but did not improved model fit (see Appendix C in Supplementary material for model specifications, output, and comparisons).

**FIGURE 3:**
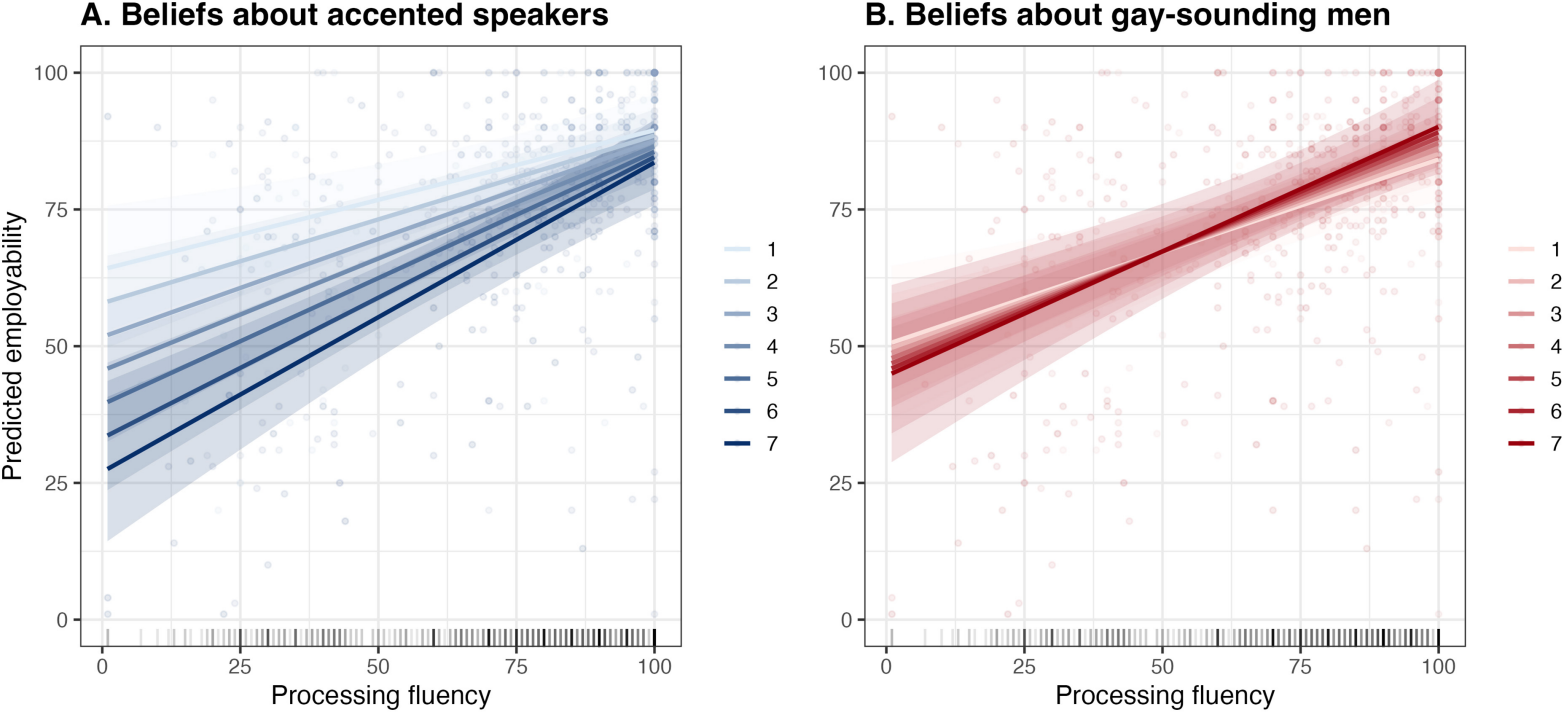
Relationship between predicted employability and processing fluency as a function of listeners’ essentialist beliefs about accented speakers **(A)** and gay-sounding men **(B)**. Solid lines represent estimated marginal means across the comprehensibility continuum, plotted separately for each level of the 7-point essentialist beliefs scale. Shaded ribbons indicate 95% CIs. Background points show raw employability ratings.

In the final model (see Appendix D in Supplementary material), processing fluency remained the strongest predictor of employability, where a 10-point decrease in processing fluency translated, on average, into a 4-point drop in speaker employability, *b* = 0.39, *SE* = 0.03, *t* = 11.90, *p* < 0.001. Furthermore, listeners’ essentialist beliefs about accented speakers predicted employability at the mean level of processing fluency, *b* = –2.30, *SE* = 0.91, *t* = –2.52, *p* = 0.013, where listeners with stronger beliefs tended to assign lower employability ratings. More importantly, essentialist beliefs also qualified the relationship between processing fluency and employability through a statistically significant interaction, *b* = 0.05, *SE* = 0.02, *t* = 2.32, *p* = 0.021, insofar as this relationship became stronger as listeners also endorsed stronger accent beliefs (Figure 3A). As processing fluency increased, the difference between listeners with high and low accent beliefs decreased; however, this moderation effect was modest in strength, consistent with its coefficient of determination (*R*^2^ = 0.01). Unlike essentialist beliefs about accented speakers, essentialist beliefs about gay-sounding men did not predict employability either as a main effect or in interaction with processing fluency (Figure 3B).

Lastly, we checked if listeners’ essentialist beliefs also accounted for processing fluency itself, considered separately from employability. A linear mixed-effects model with processing fluency as the outcome variable revealed no significant effects for essentialist beliefs about accented speakers, *b* = –1.24, *SD* = 1.10, *t* = –1.12, *p* = 0.264, or gay-sounding men, *b* = 0.15, *SD* = 1.25, *t* = 0.12, *p* = 0.908. In summary, outside of employability ratings, listeners’ essentialist beliefs contributed little to explaining processing fluency. Instead, listeners seemed to draw on their essentialist beliefs about accented speakers to qualify how much their subjective experience of processing fluency mattered for their ratings of speaker employability (Figure 3A).

## 5 Discussion

Building on a prior analysis of processing fluency as a source of bias in listener-judged employability (Teló et al., 2026), we examined if listeners’ essentialist beliefs about accented speakers and gay-sounding men moderated the relationship between processing fluency and employability. We showed that essentialist beliefs about accents, but not about sounding gay, moderated this relationship. Listeners who endorsed stronger beliefs that accents are diagnostic of other speaker qualities and that speakers can choose to modify or eliminate their accents tended to rely more heavily on their subjective experience of processing difficulty when judging speaker employability.

### 5.1 Processing fluency as a socially interpreted experience

People interpret the same experience differently, drawing on their beliefs, expectations, and assumptions to make sense of the world (Unkelbach and Greifeneder, 2013; Winkielman et al., 2025). This knowledge—collectively described as lay or naïve theories—influences how people interpret information, explain outcomes, assign responsibility, and evaluate others (Molden and Dweck, 2006). Within this perspective, voice-based essentialism can be understood as a lay theory about language and social categories (Fasoli et al., 2021; Hansen, 2020). Accordingly, listeners’ beliefs about sounding accented or gay can function as a framework or lens through which processing experiences acquire social meaning.

The idea that the role of processing fluency in people’s decision-making depends on the interpretation that they ascribe to their subjective experience is not new. Indeed, the same experience of processing difficulty can lead to different judgments depending on which interpretation (or lay theory) is accessible to or endorsed by the individual (Unkelbach and Greifeneder, 2013; Schwarz et al., 2021; Winkielman et al., 2025). For instance, theories of intelligence can moderate how processing fluency relates to perceived comprehension of texts, where people who endorse beliefs that intelligence is limited tend to interpret effortful reading (processing disfluency) as a sign that they are approaching the limits of their ability and report lower perceived comprehension (Miele and Molden, 2010). In a speech-related example (Montgomery and Acheme, 2022), listeners’ inferences about a speaker’s motives, such as whether the speaker wishes to speak clearly, amplify the relationship between processing fluency and social evaluations. A parallel dynamic appears in Dragojevic and Gasiorek (2023), where listeners’ assumption of performing well on a difficult listening task was associated with greater processing fluency and more favorable speaker evaluations, suggesting that conceptually driven mental frameworks can reshape the fluency experience itself. The present findings show that individual differences in essentialist beliefs about accented speakers may provide a conceptual framing for listeners to interpret processing fluency in relation to social judgment. For processing fluency to affect judgment, people must not only experience it but also interpret it through available theories or learned associations (Schwarz et al., 2021; Winkielman et al., 2025).

Our results imply that processing disfluency is an experiential pathway leading to less favorable evaluations of accented L2 speakers (e.g., Dragojevic et al., 2017) and that lay theories about accented speakers help explain how processing disfluency relates to downstream speaker competence (employability) judgments. As in prior research (e.g., Miele and Molden, 2010), our listeners experienced similar levels of processing fluency regardless of their essentialist beliefs, which did not predict processing fluency outside employability judgments. Put differently, listeners’ essentialist beliefs about accents did not change how easy or difficult the speakers were to understand, but these beliefs qualified the extent to which listeners’ processing fluency colored their judgment of speaker employability. The moderation was most pronounced at low levels of processing fluency, where listeners with stronger essentialist beliefs assigned markedly lower employability ratings than those with weaker beliefs (Figure 3A). This gap narrowed as processing fluency increased; in fact, at high processing fluency, listeners’ beliefs became essentially irrelevant to their employability judgment. Essentialist beliefs thus seem particularly consequential when speech is difficult to process—a situation where listeners may draw on lay theories to make sense of their experience, translating momentary comprehension difficulties into inferences about the speaker (e.g., Montgomery and Acheme, 2022; Winkielman and Schwarz, 2001). Similar to how direct evidence of a speaker’s competence can attenuate accent bias in professional evaluations (Levon et al., 2021), when speech is easy to understand, there is less interpretive pressure, and essentialist beliefs may have less purchase on the judgment.

### 5.2 Accent beliefs

Listeners who more strongly believed that accents are diagnostic of other speaker traits and that speakers can control or eliminate their accents showed a stronger relationship between processing fluency and employability. A direct (main) effect for accent beliefs also emerged, indicating that listeners with stronger beliefs tended to assign lower employability ratings on average, independent of how comprehensible they found individual speakers. While the source of this effect cannot be ascertained, it likely reflects accent-triggered stereotypes. For instance, listeners who endorse beliefs about accent being a valid diagnostic cue tend to assign lower employability ratings to L2 speakers (Hansen, 2020). Similarly, if L2 speakers are considered in control over their pronunciation, they may suffer negative consequences associated with being perceived as willingly holding onto a stigmatized identity (Gluszek and Dovidio, 2010).

Our work fits within literature demonstrating that accents function as strong indexical cues through which listeners infer various speaker characteristics, in manifestations of broader language ideologies that position some ways of speaking as normative and others as inadequate (Dragojevic et al., 2016; Preston, 2013). These ideologies often include beliefs about effort, control, and responsibility, such that accented speech is frequently interpreted through deficit-oriented frameworks that assume speakers could and perhaps should modify their pronunciation to approximate a perceived norm (Creese and Wiebe, 2012; Lippi-Green, 2012; Subtirelu, 2015). This logic maps onto the dimensions captured through our accent essentialism measure, which combined beliefs about accent being diagnostic of a person’s identity with beliefs that accent is controllable or eliminable. Within this framework, an experience of processing disfluency may become socially consequential because listeners interpret comprehension difficulty not as a situational or relational phenomenon but as evidence of speaker-specific shortcomings (Dragojevic and Giles, 2016; Gluszek and Dovidio, 2010; Hansen, 2020).

In contrast, listeners’ essentialist beliefs about gay-sounding men did not moderate the relationship between processing fluency and employability, nor did they predict employability ratings as a main effect. In our dataset, speakers’ voice-signaled sexual orientation did not generate a reliable difference in listeners’ processing fluency (Teló et al., 2026), suggesting that gay-signaling vocal cues did not introduce the kind of processing variability that essentialist beliefs could plausibly amplify. The absence of direct effects for gay-focused essentialism stands in contrast to the broader literature on sexual orientation bias (e.g., Haslam and Levy, 2006), where gay and gay-sounding men tend to receive lower workplace-relevant ratings (Fasoli et al., 2017; Taylor and Raadt, 2021). One reason is that our measure targeted voice-based essentialist beliefs, which may not capture the broader attitudinal dimensions that predict discrimination. Another possibility is that the discrimination documented in prior literature operates primarily through stereotype content (Fasoli et al., 2017; Hajek and Giles, 2005), but because the gay-signaling vocal cues did not reliably alter listeners’ processing experience in our study, essentialist beliefs had no discernible range of processing experience to map onto.

### 5.3 Limitations and future work

In terms of limitations, we did not explore the moderating effect of essentialist beliefs experimentally. Therefore, it is possible that listeners who differed in their beliefs also differed in other key variables (e.g., familiarity with the professional contexts illustrated in the recordings, experience with phonetic diversity) relevant to accent-based stereotyping (Stocker, 2017; Teló et al., 2024). Similarly, the essentialist beliefs measures were administered after listeners completed the rating task, leaving open the possibility that listeners’ judgments of employability influenced their subsequent endorsement of essentialist beliefs rather than the reverse. Longitudinal or experimental research is needed to establish the directionality of this relationship. Next, our accent essentialism measure combined beliefs about diagnosticity and controllability into a single composite score. Although those dimensions showed acceptable internal consistency, they are conceptually distinct, in that a listener can believe that accents are highly diagnostic of speaker characteristics while also viewing accents as immutable, with potentially different implications for how processing disfluency is interpreted. Disaggregating these dimensions could clarify whether diagnosticity and controllability beliefs contribute independently to processing fluency effects on judgments. Regarding listeners’ judgments of employability, they arguably reflected broad perceptions about competence or occupational suitability given that our listeners were self-selected from the general (non-specialist) population. Our findings should therefore be interpreted as evidence of stereotype-driven socio-professional evaluation rather than a direct estimate of bias in real-world hiring. Finally, our speaker pool included exclusively men. Even though listeners also make sexual orientation judgments about women’s voices (Fasoli et al., 2017), the present results might not extend to other gender groups. Similarly, speakers’ L2 accent reflected a specific variety (Spanish-accented English), so it remains an open empirical question whether the moderating role of essentialist beliefs generalizes across accents occupying different positions within global linguistic hierarchies. Naturally, “L2-accented” and “gay-sounding” are broad categories which, as perceptual dimensions, are operationalized only in relation to a particular speaker and listener sample, so our findings are necessarily specific to the speaker and listener characteristics captured here.

## 6 Conclusion

We examined whether listeners’ essentialist beliefs about accented speakers and gay-sounding men help explain when listeners’ processing experience carries more or less weight in their employability judgments of gay- and straight-sounding L1 and L2 speakers. We provided exploratory evidence that essentialist beliefs about accent (particularly that accents are diagnostic of underlying speaker characteristics and are amenable to speaker control) contribute to a stronger link between processing disfluency and employability ratings. When listeners treat accent as stable and personally attributable, they appear to more readily convert a momentary processing difficulty into an employability penalty for speakers.

Our findings generally align with frameworks in which language attitudes reflect the dual, combined influence of social stereotyping and subjective experience (Dragojevic and Dayton, 2025; Dragojevic et al., 2025), highlighting processing fluency as an experiential pathway to biased evaluations of L2 speakers, including in employment-related contexts (Dragojevic et al., 2017; Roessel et al., 2019; Teló et al., 2026). Essentialist beliefs may be one factor explaining when or for whom this experiential pathway carries greater weight in shaping social judgments (e.g., Molden and Dweck, 2006; Winkielman et al., 2025). Practically speaking, listeners might benefit from bias-reduction initiatives addressing some of their maladaptive interpretive lenses (Fry et al., 2020; Miao et al., 2025), for instance, by targeting how people interpret accent-related processing difficulty and attribute it to speakers. Ultimately, our work motivates future exploration of the mechanisms linking processing fluency to social judgment and behavior with the goal of identifying modifiable listener variables contributing to accent-based discrimination.

## Data Availability

The datasets presented in this study can be found in online repositories. The names of the repository/repositories and accession number(s) can be found below: Open Science Framework: https://doi.org/10.17605/OSF.IO/9P3HQ.
