# Supplementary material for: Essentialist beliefs about accented speakers moderate the effect of processing fluency on employability ratings
Source: Front Psychol. 2026 May 19;17:1834790. doi: 10.3389/fpsyg.2026.1834790 (PMC13226203; doi:10.3389/fpsyg.2026.1834790)
Supplement: Supplementary file 1 [file Supplementary_file_1.pdf]

Supplementary Material

Appendix A: Additional listener background information 2

Appendix B: Essentialist beliefs scales 4

Appendix C: Data modeling 6

Appendix D: Final model 8

Appendix A: Additional listener background information

We recruited 192 listeners (M_{age} = 34, *SD* = 12, range = 18–71) through local Facebook groups and Reddit/Nextdoor boards, posters placed on and off campus, and word of mouth. They had to meet two criteria: age (above 18) and residence (Calgary). We additionally used participation quotas to create a sample matching Calgary's population (Statistics Canada, 2023) in the proportion of residents born in Canada (64% in our sample, 65% in Calgary) and outside of Canada (36% in our sample, 33% in Calgary). The sample was otherwise self-selected, meaning that all participants volunteered to participate. Of the 192 listeners, 62% self-identified as women, 35% as men, and 3% as non-binary; 70% reported being heterosexual, 3% chose not to disclose, while 27% described themselves as asexual, bisexual, gay, lesbian, or queer, with the most common response being bisexual (16%). Two listeners were transgender, while the remaining (save one who chose not to disclose) were cisgender. Of the 192 listeners, 37% reported Calgary as their hometown, 27% hailed from another Canadian city, while the remaining 36% were born outside Canada. They had previously completed various degrees (27% high school, 49% undergraduate, 24% postgraduate) and described themselves politically as left (28%), moderate left (27%), center (29%), moderate right (11%), or right (5%), though 33 listeners opted not to provide their political leaning.

In terms of ethnolinguistic background, all but two listeners answered a multiple-choice (select all that apply) question, most commonly reporting "European" (52%), followed by "South Asian" (8%), "Indigenous" (7%), and nine other ethnicities or their combinations (33%). English was the most common first language (69%), followed by English and another language (4%), Portuguese (4%), Spanish (4%), and 21 other languages (19%). Most listeners (57%) indicated being proficient in one language, followed by two (31%), three (9%), and four (3%) languages. They mainly used English for daily communication (*M* = 90%, *SD* = 15, range = 11–100) and were familiar with

second language-accented English (*M* = 7.81, *SD* = 1.53, range = 1–9, where 1 = *not familiar at all*, 9 = *very familiar*).

To characterize listeners in terms of potential underlying group-based biases, we asked them to respond to statements targeting racial prejudice (four items, α = .74, adapted from Akrami et al., 2000) and homonegativity (four items, α = .68, adapted from Morrison & Morrison, 2002) through a 5-point scale (1 = *strongly disagree*, 5 = *strongly agree*). In terms of racial prejudice (e.g., “Discrimination against immigrants is still a problem in Canada,” “A multicultural Canada would be good”), listeners tended to reject ethnoracial prejudice (*M* = 4.29, *SD* = 0.72, range = 1.50–5.00). As for homonegativity (e.g., “Gay men don’t have all the rights they need,” “Even in today’s tough economic times, Canadians’ tax dollars should be used to support gay men’s organizations”), they were supportive of gay men (*M* = 3.82, *SD* = 0.88, range = 1.50–5.00). As a group, listeners therefore expressed positive perceptions of ethnoracial diversity and gay men.

To account for any stable, listener-based source of variability, we fit random intercepts for listeners while modeling the data.

Akrami, N., Ekehammar, B., & Araya, T. (2000). Classical and modern racial prejudice: A study of attitudes toward immigrants in Sweden. *European Journal of Social Psychology*, *30*(4), 521–532. https://doi.org/10.1002/1099-0992(200007/08)30:4<521::AID-EJSP5>3.0.CO;2-N

Morrison, M. A., & Morrison, T. G. (2002). Development and validation of a scale measuring modern prejudice toward gay men and lesbian women. *Journal of Homosexuality*, *43*(2), 15–37. https://doi.org/10.1300/J082v43n02_02

Appendix B: Essentialist beliefs scales

Essentialist beliefs about accented speakers were assessed using six items adapted from Hansen (2020). Responses were recorded on a 7-point Likert scale (1 = *strongly disagree*, 7 = *strongly agree*). The scale demonstrated acceptable internal consistency (Cronbach's α = .77).

1. The strength of an accent in one's speech is a sign of their personality.
2. From someone's strong or weak accent one can infer many things about the speaker.
3. It is possible to tell how someone will act by hearing their accent.
4. The type of accent in a person's speech is an important trait.
5. An accent is something that is learned, so one can change it if necessary.
6. People are capable of eliminating their accent.

Hansen, K. (2020). Accent Beliefs Scale (ABS): Scale development and validation. *Journal of Language and Social Psychology*, *39*(1), 148–171. https://doi.org/10.1177/0261927X19883903

Essentialist beliefs about gay-sounding speakers were assessed using six items adapted from Fasoli et al. (2021). Responses were recorded on a 7-point Likert scale (1 = *strongly disagree*, 7 = *strongly agree*). The scale demonstrated acceptable internal consistency (Cronbach's α = .69).

1. Whether or not a man will likely acquire gay voice is pretty much set early on in childhood.
2. Gay people can choose to sound gay or straight depending on the situation.
3. People who sound gay do so because they to emphasize their sexual orientation.
4. People who sound gay do so because they want everyone to notice immediately that they are gay.
5. A voice coach or occupational therapist can help most gay people to change their voices to sound less gay.

6. When listening to a person it is possible to detect his sexual orientation from his voice very quickly.

Fasoli, F., Hegarty, P., & Frost, D. M. (2021). Stigmatization of “gay-sounding” voices: The role of heterosexual, lesbian, and gay individuals’ essentialist beliefs. *British Journal of Social Psychology*, *60*(3), 826–850. https://doi.org/10.1111/bjso.12442

Appendix C: Data modeling

We first focused on the binary variables of speaker status (Status) and sexual orientation (SO) as predictors of employability in a baseline model (illustrated in Figure 1 the article).

Baseline model specification and output: Employability ~ Status + SO + (1 | Speaker) + (1 | Listener)

Fixed effects	Estimate	*SE*	*t*	*p*
Intercept	81.00	2.19	36.93	< .001
Speaker status (L2 vs. L1)	–7.31	2.49	–2.93	.028
Speaker SO (gay vs. straight)	–1.28	1.54	–0.83	.407
Random effects	Variance	*SD*		
Speaker	9.15	3.02		
Listener	154.53	12.43		
Residual	233.91	15.29		

Note. Reference levels: L1 for speaker status, straight for speaker sexual orientation. Marginal R^2 = .03; conditional R^2 = .43.

We then updated this model (stepwise) with listener ratings of accentedness ($\Delta R^2_{\text{marginal}}$ = .03), processing fluency ($\Delta R^2_{\text{marginal}}$ = .17), and their essentialist beliefs ($\Delta R^2_{\text{marginal}}$ = .02).

Main-effects model specification and output: Employability ~ Status + SO + Accentedness + Processing Fluency + Accent Beliefs + Gay-Sounding Beliefs + (1 | Speaker) + (1 | Listener)

Fixed effects	Estimate	*SE*	*t*	*p*
Intercept	78.10	2.09	37.33	< .001
Speaker status (L2 vs. L1)	0.07	2.63	0.03	.979
Speaker SO (gay vs. straight)	–2.71	1.39	–1.95	.052
Accentedness	–0.03	0.03	–0.92	.356

Processing fluency	0.40	0.03	12.06	< .001
Accent beliefs	–2.44	0.90	–2.69	.008
Gay-sounding beliefs	0.47	1.03	0.46	.645
Random effects	Variance	*SD*		
Speaker	8.95	2.99		
Listener	99.55	9.98		
Residual	190.17	13.79		

Note. Reference levels: L1 for speaker status, straight for speaker sexual orientation. Marginal R^2 = .25; conditional R^2 = .52.

Appendix D: Final model

Addressing our main question, two-way interaction terms between listeners' processing fluency and essentialist beliefs were added, which improved model fit relative to the main-effects model, $\chi^2(2) = 11.18$, $p = .004$, and explained an additional 1% of employability ratings, for a final model explaining 26% of the variance ($R^2_{conditional} = .54$; 620 datapoints from 182 valid listeners). Final model specification and output: Employability ~ Status + SO + Accentedness + Accent Beliefs × Processing Fluency + Gay-Sounding Beliefs × Processing Fluency + (1 | Speaker) + (1 | Listener)

Fixed effects	Estimate	*SE*	*t*	*p*
Intercept	78.66	2.02	38.98	< .001
Speaker status (L2 vs. L1)	–0.50	2.50	–0.20	.848
Speaker SO (gay vs. straight)	–3.05	1.37	–2.22	.027
Accentedness	–0.03	0.03	–1.13	.257
Processing fluency	0.39	0.03	11.90	< .001
Accent beliefs	–2.30	0.91	–2.52	.013
Gay-sounding beliefs	0.50	1.04	0.48	.634
Accent beliefs × Processing fluency	0.05	0.02	2.32	.021
Gay-sounding beliefs × Processing fluency	0.02	0.03	0.68	.499
**Random effects**	**Variance**	***SD***		
Speaker	7.67	2.77		
Listener	104.62	10.23		
Residual	184.92	13.60		

Note. Reference levels: L1 for speaker status, straight for speaker sexual orientation. Marginal R^2 = .26; conditional R^2 = .54.

We also examined whether the moderating role of essentialist beliefs depended on speakers' status (L1 vs. L2, gay vs. straight). Higher-order interactions among Accent Beliefs × Processing Fluency × Language Status and Gay-Sounding Beliefs × Processing Fluency × Sexual Orientation were tested. A likelihood ratio suggested that these interactions did not improve model fit and were therefore not retained, $\chi^2(6) = 2.33$, $p = .887$.
